# Promoter mutagenesis for fine‐tuning expression of essential genes in *Mycobacterium tuberculosis*

**DOI:** 10.1111/1751-7915.12875

**Published:** 2017-10-27

**Authors:** Francesca Boldrin, Giulia Degiacomi, Agnese Serafini, Gaëlle S. Kolly, Marcello Ventura, Claudia Sala, Roberta Provvedi, Giorgio Palù, Stewart T. Cole, Riccardo Manganelli

**Affiliations:** ^1^ Department of Molecular Medicine University of Padova 35121 Padova Italy; ^2^ Ecole Polytechnique Fédérale de Lausanne Global Health Institute Station 19 1015 Lausanne Switzerland; ^3^ Department of Biology University of Padova 35121 Padova Italy; ^4^ Present address: Mycobacterial Metabolism and Antibiotic Research Laboratory The Francis Crick Institute 1 Midland Road London NW1 1AT UK; ^5^ Present address: Institut Pasteur de Lille 1, rue du professeur Calmette 59019 Lille Cedex France

## Abstract

A range of regulated gene expression systems has been developed for mycobacteria in the last few years to facilitate the study of essential genes, validate novel drug targets and evaluate their vulnerability. Among these, the TetR/Pip‐OFF repressible promoter system was successfully used in several mycobacterial species both *in vitro* and *in vivo*. In the first version of the system, the repressible promoter was P_*ptr*_, a strong Pip‐repressible promoter of *Streptomyces pristinaespiralis*, which might hamper effective downregulation of genes with a low basal expression level. Here, we report an enhanced system that allows more effective control of genes expressed at low level. To this end, we subjected P_*ptr*_ to targeted mutagenesis and produced 16 different promoters with different strength. Three of them, weaker than the wild‐type promoter, were selected and characterized showing that they can indeed improve the performances of TetR/Pip‐OFF repressible system both *in vitro* and *in vivo* increasing its stringency. Finally, we used these promoters to construct a series of bacterial biosensors with different sensitivity to DprE1 inhibitors and developed a whole‐cell screening assay to identify inhibitors of this enzyme.

## Introduction

Tighty regulated gene expression systems are powerful tools widely used to study essential genes, validate drug targets and evaluate their vulnerability (Schnappinger, 2015). In recent years, several such systems were developed for mycobacteria [reviewed in (Schnappinger and Ehrt, 2014; Choudhary *et al*., 2016)]. The most widely used are those based on the TetR repressor (Carroll *et al*., 2005; Ehrt *et al*., 2005), but recently other systems based on CRISPR interference (Choudhary *et al*., 2015; Singh *et al*., 2016), on the theophylline riboswitch (Seeliger *et al*., 2012) or on dual‐control switches which combine transcriptional regulation and regulated proteolysis (Kim *et al*., 2011, 2013) were shown to be highly effective.

In previous work, we reported the development of a repressible promoter system (TetR/Pip‐OFF) based on two chromosomally encoded repressors and the tunable promoter P_*ptr*_ (Boldrin *et al*., 2010). In this system, entirely integrated in single copy in the mycobacterial genome, the gene encoding the tetracycline‐responsive repressor TetR is constitutively expressed, while the gene coding for the pristinamycin‐responsive repressor Pip is expressed from a TetR‐dependent promoter, and the gene of interest is expressed from P_*ptr*_, a strong promoter repressible by Pip. In the absence of ATc, TetR represses *pip* expression and the target gene is expressed; in the presence of ATc, TetR releases the promoter of the Pip gene resulting in repression of P_*ptr*_. The system can be further modulated by the addition of pristinamycin I, which can alleviate P_*ptr*_ repression by Pip (Boldrin *et al*., 2010).

Since then, the TetR/Pip‐OFF system has been successfully used by several groups to study essential genes and validate drug targets *in vitro* and *in vivo* in different mycobacterial species (Serafini *et al*., 2009, 2013; Cortes *et al*., 2011; Di Luca *et al*., 2012; Mondino *et al*., 2013; Ventura *et al*., 2013; Ahmed *et al*., 2014, 2016; Bazet Lyonnet *et al*., 2014; Bhowmick *et al*., 2014; Boldrin *et al*., 2014; Kolly *et al*., 2014a,b; Pandey and Rodriguez, 2014; Verma and Chatterji, 2014; Gola *et al*., 2015; Gupta *et al*., 2015; Mori *et al*., 2015; Hu *et al*., 2016; Degiacomi *et al*., 2017). This broad experience taught us that the main drawback of this otherwise very succesful system was the strength of the P_*ptr*_ promoter, which causes overexpression of the gene of interest, when this is physiologically expressed at low level, with resulting accumulation of its product. As a consequence of such accumulation, the effect of gene repression in these cases was often visible only after a number of serial passages of the bacterial culture in the presence of the repressor (ATc). This step was needed to titrate down the protein product. Moreover, we failed to obtain a phenotype in conditional mutants of genes expressed at very low levels (such as transcriptional regulators), probably because even when repressed their residual expression, due to promoter leakage, produced sufficient target protein to effect its physiological function (unpublished data). In this manuscript, we describe the construction and characterization of a series of P_*ptr*_ promoter mutants of different strength and show that these promoters can be used in the context of the Tet/Pip‐OFF repressible system thus overcoming the problems outlined above.

## Results

### Promoter mutagenesis

**Figure 1:**
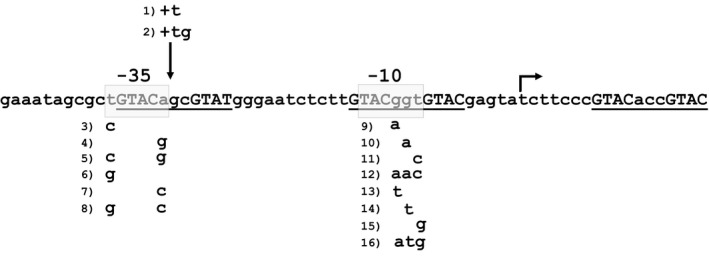
Mutations introduced in the P_*ptr*_ promoter. Putative ‐10 and ‐35 are boxed, while the three Pip‐binding regions are underlined. Conserved repeats within the Pip‐binding regions are in capital letters. Mutations are numbered (1–16), and the nucleotides replacing the original are shown below the wt sequence. Mutations 1 and 2 are insertions; the vertical arrow indicates the point where nucleotide(s) was/were inserted. The transcriptional start site is indicated by an arrow.

The TetR/Pip‐OFF repressible promoter system has been successfully used to construct several conditional mutants in *Mycobacterium tuberculosis* (Boldrin *et al*., 2010). However, the high strength of the P_*ptr*_ promoter, on which the system is based, may represent a problem when the target gene is physiologically expressed from a weak promoter. To solve this problem, we designed a mutagenic strategy to weaken the promoter by introducing point mutations in the ‐10 or ‐35 consensus sequences or by modifying the length of the spacer region. Using PCR, we obtained eight promoters with mutations in the ‐10 region, six promoters with mutations in the ‐35 region and two promoters where the length of the spacer region was modified (Fig. 1). Mutated and wt promoters were cloned upstream of a promotorless *egfp* gene in the integrative plasmid pFRA61, containing the TetR/Pip‐OFF system (Boldrin *et al*., 2010). The resulting plasmids were transformed in both *Mycobacterium smegmatis* mc^2^155 and in *M. tuberculosis* H37Rv (Table S1).

### Characterization of the mutant promoters

**Figure 2:**
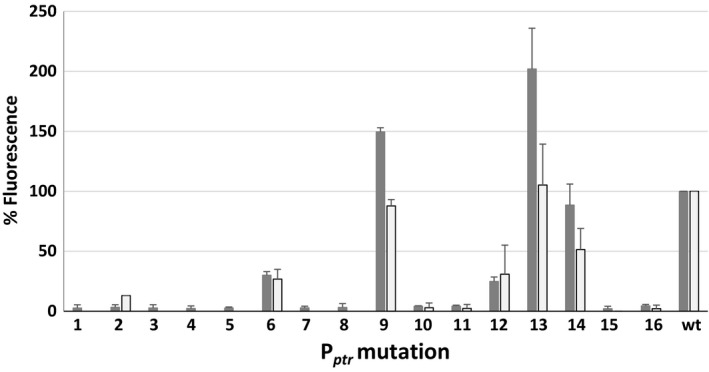
Relative fluorescence of *Mycobacterium tuberculosis* (dark grey) and *M. smegmatis* (light grey) strains harbouring integrative plasmids encoding the *egfp* gene downstream of the different P_*ptr*_ promoter mutants (1–16). Fluorescence of the strain expressing *egfp* from wt P_*ptr*_ was considered as 100%. For unknown reasons, we could not introduce the plasmids containing the P_*ptr*_ mutations 5 and 8 in *M. smegmatis*.

**Figure 3:**
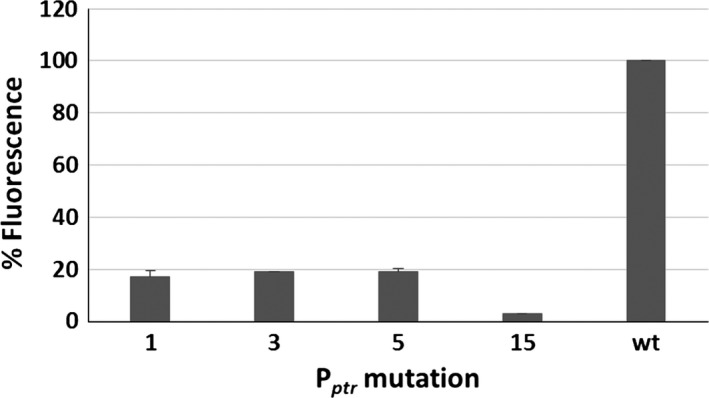
Relative fluorescence of *Mycobacterium tuberculosis* strains harbouring replicative plasmids encoding the *egfp* gene downstream P_*ptr*_ promoters shown to have undetectable activity in Fig. 2. Fluorescence of the strain expressing *egfp* from wt P_*ptr*_ was considered as 100%.

To evaluate promoter strength, *M. tuberculosis* and *M. smegmatis* strains harbouring the integrative plasmids containing the *egfp* transcriptional fusions were grown to exponential phase, and their fluorescence was measured using a fluorometer. Results clearly showed that the mutations resulted in promoters with a wide range of strength and that almost all of the new promoters behaved similarly in *M. tuberculosis* and in *M. smegmatis* (Fig. 2). Interestingly, mutations in the spacer region and in the ‐35 consensus sequence always caused strong attenuation of the promoter, whereas mutations in the ‐10 consensus sequence resulted in partial attenuation or even an increase in promoter activity (P_*ptr9*_, P_*ptr13*_, P_*ptr14*_). Some promoters showed an activity which was barely over the background. To determine whether their activity was totally abrogated or so low as to be undetectable when present in a single chromosomal copy, some of them were subcloned together with the *egfp* gene in a replicative plasmid and again introduced in *M. tuberculosis*. Figure 3 reports the relative fluorescence of the resulting strains, indeed confirming that in these conditions their activity was detectable.

**Figure 4:**
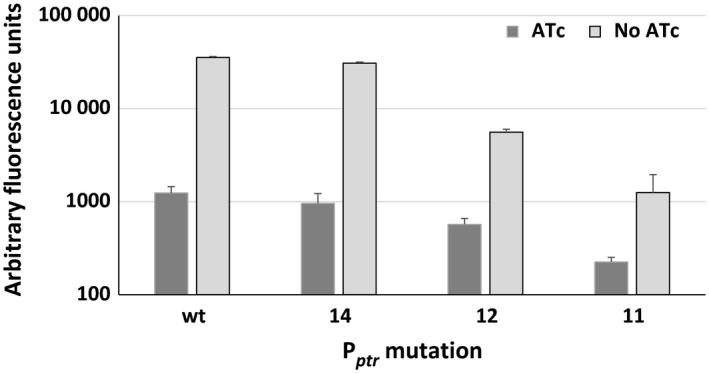
Fluorescence of *Mycobacterium tuberculosis* strains harbouring integrative plasmids encoding the *egfp* gene downstream of different P_*ptr*_ promoter mutants grown with or without ATc.

Starting from these data, we chose three promoters with different relative strength for further characterization: P_*ptr11*_, P_*ptr12*_ and P_*ptr14*_. To define whether these promoters were still repressible by Pip, the *M. tuberculosis* strains containing transcriptional fusions to *egfp* were grown to exponential phase with or without ATc 500 ng ml^−1^, and fluorescence was measured. As shown in Fig. 4, all of them were still repressible and suitable for use in the TetR/Pip‐OFF system.

### Construction and characterization of conditional mutants expressing dprE1 from promoters with different strength

Previously, we reported the construction and characterization of a *dprE1* conditional mutant based on the TetR/Pip‐OFF system (Kolly *et al*., 2014a). We demonstrated that *dprE1* is an essential gene, as its repression upon exposure to ATc resulted in growth arrest. However, the strain had to be subcultured several times in the presence of ATc to deplete the gene product effectively. This was due to the higher strength of P_*ptr*_ with respect to the physiological promoter of *dprE1*, which resulted in its overexpression in the conditional mutant (Kolly *et al*., 2014a). To rectify this, we constructed three conditional mutants expressing *dprE1* from P_*ptr11*_, P_*ptr12*_, and P_*ptr14*_ (TB386, TB387, and TB388 respectively; Table 1). The three conditional mutants were grown in parallel with the original conditional mutant TB110 expressing *dprE1* from the wt P_*ptr*_ in the presence of ATc 500 ng ml^−1^. As shown in Table 2, the number of passages needed to observe the growth arrest was directly correlated to the strength of the promoter.

**Table 1:** *dprE1* conditional mutants based on the TetR/Pip‐OFF or the Pip‐ON systems

Mutation	PlasmidTetR/Pip‐OFF	Strain	Plasmid Pip‐ON	Strain
11	pFRA184	TB386	pFRA212	TB434
12	pFRA186	TB387	pFRA213	TB435
14	pFRA188	TB388	pFRA214	TB436

**Table 2:** Passages in fresh ATc needed to stop the growth of dprE1 conditional mutants

Strains	Pptr strength^a^ (%)	Passages in ATc 500 ng ml^−1^ b
TB386	4	3
TB387	25	4
TB388	89	7
TB110	100	> 7

a Referred to P_*ptr*_ wt which was considered as 100%.

b Strains were grown in standing condition with or without ATc. Every 72–96 h, bacteria were diluted into fresh media containing ATc.

**Figure 5:**
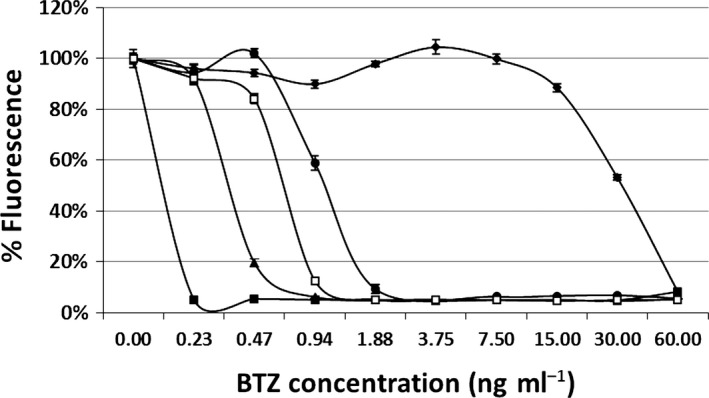
Resazurin microplate assay (REMA) to determine the BTZ sensitivity of *Mycobacterium tuberculosis* conditional mutants expressing *dprE1* from different promoters. Filled squares, TB386 (P_*ptr11*_); empty squares, H37Rv; triangles, TB387 (P_*ptr12*_); circles, TB388 (P_*ptr14*_); diamonds, TB110 (P_*ptr*_).

**Figure 6:**
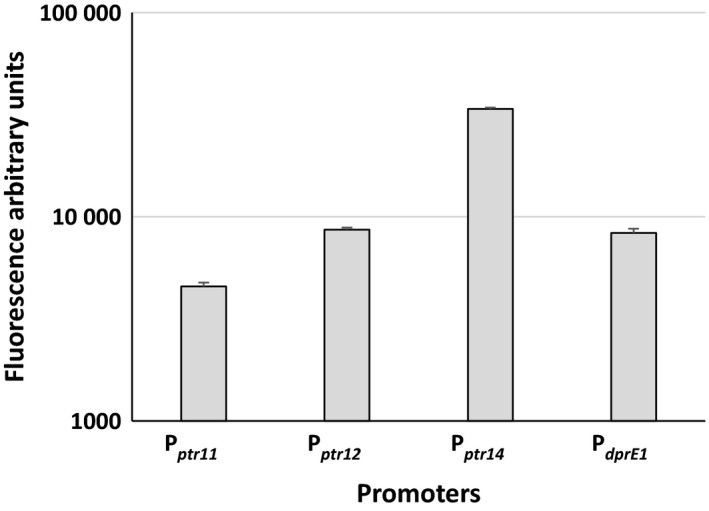
Fluorescence of *Mycobacterium tuberculosis* strains harbouring integrative plasmids encoding the *egfp* gene downstream of the different P_*ptr*_ promoter mutants and of the *dprE1* promoter.

The protein encoded by *dprE1* is the target of benzothiazinone (BTZ), a new antimycobacterial compound currently under development (Makarov *et al*., 2009, 2014; Piton *et al*., 2017). As the level of DprE1 has been shown to correlate with the sensitivity to BTZ, we measured the sensitivity of the new conditional mutants to this molecule: as illustrated in Fig. 5, there was a clear correlation between the level of promoter activity and the MIC to BTZ. The strain expressing *dprE1* from P_*ptr12*_ (TB387) showed the same MIC for BTZ as H37Rv, suggesting that in these two strains, *dprE1* is expressed at a similar level and that P_*ptr12*_ and the physiological promoter of *dprE1* are similarly active (Table 3). To confirm this hypothesis, the *dprE1* natural promoter was cloned upstream of a promoterless *egfp* gene in the same integrative plasmid used to evaluate the P_*ptr*_ mutants. The resulting plasmid was introduced into *M. tuberculosis* to obtain TB419, whose fluorescence was compared with that of the *M. tuberculosis* strain expressing *egfp* from P_*ptr11*_, P_*ptr12*_, or P_*ptr14*_. Results in Fig. 6 confirmed that the strength of the *dprE1* promoter was comparable to that of P_*ptr12*_.

**Table 3:** BTZ MIC85 obtained in the experiment shown in Fig. 5 compared with promoter strength

Strain	Promoter strength (%)	MIC_85_ (ng ml^−1^)
TB386	4	0.23
TB387	25	0.94
TB388	89	1.88
TB110	100	60

**Figure 7:**
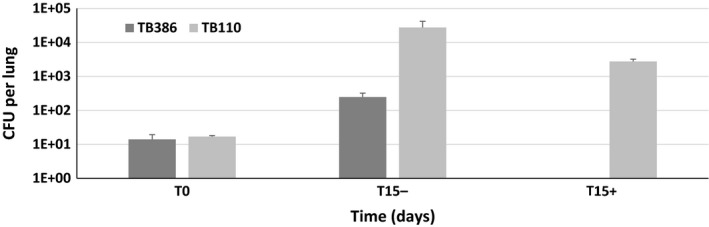
Bacterial burden of TB110 and TB386 in mouse lungs. One group of mice was treated with doxycycline (+) from the beginning of the experiment. Mice were sacrificed at time 0 and 15 days after infection.

Finally, the *M. tuberculosis* conditional mutants expressing *dprE1* from wt P_*ptr*_ (TB110) or from P_*ptr11*_ (TB386) were used to infect two groups of mice, one of which was treated with doxycycline. As visible in Fig. 7, at day 15 post‐infection, mice infected with TB110 showed a higher bacterial burden than those infected with TB386 suggesting that expression of *dprE1* from P_*ptr11*_ results in suboptimal intracellular DprE1 concentration for *in vivo* growth. Moreover, 15 days after infection treatment with doxycycline resulted in a 10‐fold decrease in the bacterial burden of TB110 and in TB386 levels decreasing below the limit of detection, thus demonstrating the essentiality of *dprE1* during infection.

### Development of a whole‐cell screening assay to identify inhibitors of DprE1

The development of whole‐cell screenings based on bacterial biosensors sensitized to inhibitors of specific targets represents a novel strategy to identify new antibacterial drugs (Hughes and Karlen, 2014; Schnappinger, 2015). As DprE1 has been shown to be a valid and vulnerable target for *M. tuberculosis* (Makarov *et al*., 2009), we decided to use our set of promoters to develop an assay to identify molecules able to inhibit its activity. To this end, we constructed a set of *dprE1* conditional mutants in which *dprE1* was controlled by P_*ptr11*_, P_*ptr12*_, or P_*ptr14*_ under the control of the Pip‐ON inducible system (Forti *et al*., 2009). These plasmids were then introduced into the conditional *dprE1* mutant TB110 by plasmid switching (Pashley and Parish, 2003) obtaining the strains TB434, TB435 and TB436 respectively (Table 1). The resulting strains were pristinamycin‐dependent, as their only functional copy of *dprE1* was under transcriptional control of one of the P_*ptr*_ promoters, which are not active in the absence of pristinamycin due to Pip‐mediated repression (Fig. S1).

**Figure 8:**
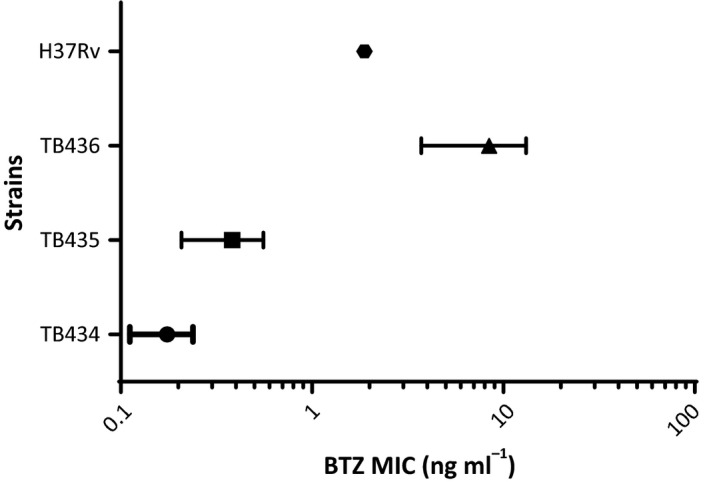
MIC range of different *Mycobacterium tuberculosis* conditional mutants in the presence of different pristinamycin concentrations.

Finally, using REMA, we determined the MIC to BTZ of these three strains grown in the presence of different concentrations of pristinamycin. As shown in Table 4 and Fig. 8, the MIC was proportional to the strength of the promoter transcribing *dprE1* and to the concentration of pristinamycin with a range going from 0.12 ng ml^−1^ (TB434, 25 ng ml^−1^ pristinamycin) to 15 ng ml^−1^ (TB436, 100 ng ml^−1^ pristinamycin), while the MIC of H37Rv was 1.88 ng ml^−1^.

**Table 4:** MIC (ng ml^−1^) to BTZ of H37Rv and different *Mycobacterium tuberculosis dprE1* conditional mutants upon the addition of different amounts of pristinamycin

Strain	Pristinamycin concentration (ng ml^−1^)				
	0	25	50	75	100
H37Rv	1.88				
TB434		0.12	0.12	0.23	0.23
TB435		0.23	0.47	0.47	0.47
TB436		3.75	7.5	7.5	15

## Discussion

The main aim of this work was to mutagenize the repressible promoter P_*ptr*_ to increase the tunable range of the TetR/Pip‐OFF repressible system (Boldrin *et al*., 2010). The *pip* promoter encloses three Pip operators each containing two directly repeated 4 bp palindromes separated by a 3 bp spacer. Two of these operators overlap the putative ‐10 and ‐35 consensus sequences, while the third is located 10 nucleotides after the *ptr* transcriptional start site (Fig. 1; Blanc *et al*., 1995). As our aim was to change P_*ptr*_ strength without interfering with its regulation by the pristinamycin‐dependent transcriptional repressor Pip, mutations were introduced either before the operators or within the spacers (Fig. 1). Two insertions increased the distance between the putative ‐10 and ‐35 sequences (mutations 1 and 2), and most of the changes in the putative ‐35 sequence (mutations 3–8) resulted in very strong attenuation of transcription. However, mutations in the putative ‐10 sequence showed a wide variation of transcriptional activity: some of them, similarly to mutations in the ‐35 sequence, caused a dramatic decrease in activity (mutations 10, 11, 15 and 16), others (mutations 12 and 14) resulted in decreased, but still appreciable level of activity, or in an increased level of activity (mutations 9 and 13). It should be noted; however, that only mutations of the first and last base of the ‐35 hexamer were designed and tested, given the overlap of this sequence with the first Pip operator (Fig. 1). Mycobacterial promoters usually have well‐conserved ‐10 regions, while ‐35 are less conserved and hardly recognizable, probably because of the high number of sigma factors operating in these bacteria (Bashyam *et al*., 1996; Newton‐Foot and Gey van Pittius, 2013; Manganelli, 2014). Moreover, several mycobacterial promoters present extended ‐10 regions (containing a TGn sequence at the 5′‐end) which increase their potency, and it was proposed that these promoters do not require the presence of a ‐35 sequence (Kenney and Churchward, 1996). It is not clear if the P_*ptr*_ promoter has an extended ‐10, as the borders of its ‐10 consensus sequence are not easily recognizable, but a TG sequence is present just before its locus (Fig. 1).

Three mutant promoters with variable strengths were selected, and the possibility for Pip to repress their activity was confirmed. To demonstrate that these promoters could be used to improve the performances of the TetR/Pip‐OFF repressible promoter system, we constructed three conditional *dprE1* mutants using these promoters and compared them to the previously characterized *dprE1* mutant obtained with wt P_*ptr*_. DprE1 is an essential decaprenylphosphoryl D‐ribose epimerase, an enzyme involved in arabinogalactan and lipoarabinomannan biogenesis (Kolly *et al*., 2014a) and is the target of BTZ, one of the most potent and best characterized candidates in the TB drug development pipeline (Makarov *et al*., 2009; Brecik *et al*., 2015). The *dprE1* conditional mutant was functional to show the essentiality of this gene, but due to the higher strength of the P_*ptr*_ promoter in comparison with its physiological promoter, several passages in the presence of ATc were needed to titrate DprE1 down and stop cells growing when cultivated without aeration (Kolly *et al*., 2014a) and data not shown). Indeed, when this mutant was cultivated in parallel with the new mutants in these conditions, it was clear that the weakest promoters required fewer passages in the presence of ATc to stop bacterial growth. Moreover, this agreed well with the MIC to BTZ of the various mutants which increased with promoter potency (and thus with DprE1 intracellular concentration; Table 3). The MIC of the conditional mutant expressing *dprE1* from P_*ptr12*_ was equal to that of the wt strain, suggesting a similar potency of these two promoters, which was confirmed when the two promoters were compared (Fig. 6). However, careful inspection of the data obtained by REMA shows that H37Rv is slightly more resistant to BTZ than the conditional mutant (Fig. 5); this may be explained either by the existence of a metabolic burden in the mutant strain due to the presence of the integrated plasmid and the TetR/Pip‐OFF system which might increase its sensitivity, or more interestingly by the possibility that in the presence of a drug inhibiting DprE1, the *dprE1* promoter might be induced thereby increasing DprE1 intracellular concentration.

When the original mutant and the mutant in which *dprE1* was transcribed from the weakest of the selected promoters (P_*ptr11*_) were used to infect mice, we proved that the effect of *dprE1* repression was more dramatic in the strain expressing *dprE1* at the lower level (more than two log reduction in the number of cfu in 2 weaks *vs* one log reduction for the other strain). Interestingly, this strain grew more slowly than the other one in the lungs even in the absence of doxycycline treatment. As the two strains grow similarly *in vitro* (not shown), this suggests that bacteria require more DprE1 expression during infection than during growth in axenic culture, probably for the faster protein turnover in this environment generated by exposure to stress. It is worth noting that as the strength of P_*ptr11*_ is lower than that of P_*dprE1*_ (Fig. 6), this strain probably contains a suboptimal concentration of DprE1 which might well explain its attenuation even in the absence of doxycyline treatment.

Finally, we wanted to investigate the possibility to use our promoters to develop bacterial biosensors sensitized to inhibitors of specific targets to optimize target‐based whole‐cell screenings. As proof of principle, we used the *dprE1* gene. As whole‐cell screenings with *M. tuberculosis* usually need long incubation times, we reasoned that ATc, which in our experience has a short half‐life, was not the molecule of choice to develop such a system. We therefore used the Pip‐ON inducible system, based on the more stable pristinamycin (Forti *et al*., 2009). In this system, the pristinamycin‐sensitive transcriptional repressor Pip is constitutively expressed thereby inhibiting transcription at P_*ptr*_, but in the presence of pristinamycin, it releases its operators in a dose‐dependent manner and allows transcription. Measurement of MIC to BTZ of the three Pip‐ON‐based conditional mutants in the presence of two different concentrations of pristinamycin clearly showed that each strain was more vulnerable to the drug when the expression of *dprE1* was lower due to a lower concentration of pristinamycin. However, the differences due to pristinamycin‐dependent *dprE1* expression modulation were smaller than those due to the strength of the different promoters. It is worth noting that the strain with the lower MIC (TB434 grown in the presence of 25 ng ml^−1^ of pristinamycin) was 16‐fold more sensitive than H37Rv, while the strain with the highest MIC (TB436 grown in the presence of 100 ng ml^−1^ of pristinamycin) was 8‐fold more resistant than H37Rv (Fig. 8). Consequently, the 128‐fold MIC range, from the most resistant to the most sensitive strain, provides a very sensitive window to screen for active compounds.

In conclusion, the construction and characterization of different P_*ptr*_ promoter mutants widely expanded the potential of Pip‐based tunable promoter systems, thus increasing the range of genes whose expression can be effectively manipulated for the construction of bacterial biosensors.

## Experimental procedures

### Strain and culture conditions

The following bacterial strains were used in this study: *Escherichia coli* Top10 (Invitrogen, Carlsbad, CA, USA), DH5α and HB101 (laboratory stock), *M. smegmatis* mc^2^155 (laboratory stock), and *M. tuberculosis* H37Rv (laboratory stock). *E. coli* strains were grown at 37°C in Luria‐Bertani (LB) broth or on LB agar plates. Mycobacterial strains were grown at 37°C in Middlebrook 7H9 broth (Difco, Franklin Lakes, NJ, USA) in 150 ml roller bottles with slow rotation (3 rpm), in 10 ml screw‐cap tubes without agitation, or 7H10 agar plates (Difco), supplemented with 0.2% glycerol and 0.05% Tween‐80. For growth of *M. tuberculosis*, the medium was supplemented with 10% albumin‐dextrose‐sodium chloride complex (ADN; Jacobs *et al*., 1991). When needed, antibiotics were added to the media at the following concentrations: streptomycin (Sm) 20 μg ml^−1^, kanamycin (Km) 50 μg ml^−1^ (*E. coli*) or 20 μg ml^−1^ (*M. smegmatis* and *M. tuberculosis*), hygromycin (Hyg) 150 μg ml^−1^ (*E. coli*) or 50 μg ml^−1^ (*M. smegmatis* and *M. tuberculosis*). Anhydrotetracycline (ATc; Sigma, St. Louis, MO, USA) or pristinamycin (Molcan Corporation, Richmond Hill, ON, Canada) were added when required at the concentrations indicated in the text. Benzothiazinone 043 (BTZ) was dissolved in DMSO at a concentration of 9 μg ml^−1^. Preparation of electrocompetent cells, electroporation and preparation of mycobacterial genomic DNA were performed as previously described (Maciag *et al*., 2007).

### Plasmid construction and PCR‐mutagenesis

The sequence encoding eGFP was PCR amplified from pMV4‐36 (Cascioferro *et al*., 2010) using primers RP1335/RP1336 (Table S2) and subcloned in the suicide vector pFRA50 downstream of the repressible promoter P_*ptr*_ (Boldrin *et al*., 2010)_._ The P_*ptr*_‐*egfp* fragment was than subcloned in TOPO (Invitrogen). This plasmid was used as a template for PCR‐mutagenesis using upper primers overlapping the P_*ptr*_ sequence and containing the different point mutations and a lower primer overlapping the final part of *egfp* (Fig. S2 and Table S2). The amplified mutagenized P_*ptr*_‐*egfp* fragments were cloned in TOPO and, after confirmation of the presence of the correct mutation by sequencing, subcloned in both pFRA61 (integrative plasmid harbouring the TetR/Pip‐OFF system; Kolly *et al*., 2014a) and in pMV261 (replicative plasmid) (Cooksey *et al*., 1993). The resulting integrative plasmids were introduced into *M. smegmatis* mc^2^155 and *M. tuberculosis* H37Rv while replicative plasmids were electroporated into *M. smegmatis* MS212 and *M. tuberculosis* TB262 (mc^2^155‐ and H37Rv‐derivatives harbouring the TetR/Pip‐OFF system). Vectors and bacterial strains are listed in Table S1.

### Construction of a dprE1‐reporter plasmid

A fragment of 518 bp immediately upstream of *dprE1* containing its putative promoter was amplified with primers RP1629/RP1630 using Phusion^®^ High‐Fidelity DNA Polymerase and cloned upstream of a promoterless *egfp* generating pFRA194. The P_*dprE1*_‐*egfp* fragment was excised from this plasmid and subcloned into the integrative plasmid pFRA61 (Kolly *et al*., 2014a) obtaining pFRA206. This plasmid was introduced in *M. tuberculosis* H37Rv to obtain TB419.

### Construction of *M. tuberculosis* dprE1 conditional mutants

The gene encoding DprE1 was amplified using primers RP806/RP807 (Table S2) using Phusion^®^ High‐Fidelity DNA Polymerase (New Englands Biolabs, Ipswich, MA, USA) and cloned into pCR‐Blunt II‐TOPO (Invitrogen). The amplified fragment was than excised and subcloned (i) into three integrative plasmids containing the TetR/Pip‐OFF system and one of the three mutated P_*ptr*_ (mutations 11, 12, and 14) to obtain pFRA183, pFRA186, and pFRA188 respectively and (ii) into three integrative plasmids containing the Pip‐ON system and one of the three mutated P_*ptr*_ (mutations 11, 12, and 14) to obtain pFRA212, pFRA213, pFRA214 respectively. These six plasmids were introduced into the *dprE1* conditional mutant TB110 (Kolly *et al*., 2014a) by plasmid switching (Pashley and Parish, 2003) generating TB386, TB387, TB388 (TetR/Pip‐OFF) and TB434, TB435, TB436 (Pip‐ON) respectively (Table 1).

### Fluorescence assay

eGFP fluorescence was measured in Nunclon 96‐well Flat Bottom Black Polystyrol FluoroNunc microplates (Thermo Scientific, Carlsbad, CA, USA) to minimize background fluorescence. Bacterial strains were inoculated in 5 ml of 7H9/ADC, supplemented with appropriate antibiotics, and grown to OD_540_ = 0.5. Cells were harvested by centrifugation (5 min, 10 000 rpm), and pellets were resuspended in a volume of 0.1 ml of Middlebrook 7H9 to obtain a concentration of about 10^9^ cfu ml^−1^. Then, 10^8^ cfu were added in the microplates, and fluorescence values were measured with Infinite 200Pro microplate reader (Tecan Group, Mannendorf, Switzerland), using excitation and emission wavelengths of 485 and 535 nm respectively. Wells containing only medium or bacterial cultures of *M. smegmatis* and *M. tuberculosis* strains not expressing GFP were used as controls to determine bacterial autofluorescence that was used to subtract the background from the measurements. All the experiment were repeated at least twice in triplicate. Data were reported as mean ± standard deviation.

### Benzothiazinone susceptibility test

Bacterial strains were grown in 3 ml of Middlebrook 7H9 supplemented with the appropriate antibiotics to OD_540_ 0.2‐0.4. Cultures were diluted to a theoretical OD_540_ of 0.0005 and 200 μl of the resulting suspension were spotted into each well of the first column of a Nunclon 96‐well Flat Bottom Black Polystyrol FluoroNunc microplates (Thermo Scientific). Starting from the second column, 100 μl of bacteria were added to the other wells of the plate. BTZ (stock concentration = 9 μg ml^−1^) was added to a final concentration of 60 ng ml^−1^ in each well of the first column and then serial dilutions (1:1) were made. Plates were incubated 7 days at 37°C, and then 10 μl of AlamarBlue (Invitrogen) was added to each well and incubated at 37°C. The detection was performed after 24 h of incubation using an Infinite 200Pro microplate reader (Tecan Group), with excitation and emission wavelengths of 535 and 590 nm respectively. The lowest drug concentration that resulted in growth inhibition of at least 85% was considered as the MIC. The experiments were repeated twice in triplicate. Data were reported as mean ± standard deviation.

### *In vivo* experiments

Female C57BL/6 mice (Charles River Laboratories, Wilmington, MA, USA) were infected by low‐dose aerosol with the *dprE1* conditional knock‐down strains. Mice were fed with doxycycline‐containing food (2000 ppm, Harlan) starting from 5 days before infection. Control groups were fed with regular diet, equal in composition to the doxycycline‐containing food except for the antibiotic. Four mice per group and time point were sacrificed at day 0 and 2 weeks post‐infection. Lung homogenates were plated on 7H10 plates supplemented with ampicillin (100 μg ml^−1^) and cycloheximide (10 μg ml^−1^). The experiment was performed once. Data were calculated as mean ± standard deviation. Experimental procedures involving animals were approved by the Swiss Cantonal and Federal Authorities (Authorization number 2658).

## Conflict of interest

None of the authors have any potential conflict of interest to declare.

## Supporting information


**Fig. S1.** Pristinamycin‐dependent growth of Pip‐ON based *dprE1* conditional mutants.

**Fig. S2.** Strategy to construct promoter mutants.

**Table S1.** Mutagenized promoters and corresponding strains.

**Table S2.** Primers used in this study.
