# Supplementary material for: Promoter mutagenesis for fine‐tuning expression of essential genes in Mycobacterium tuberculosis
Source: Microb Biotechnol. 2017 Oct 27;11(1):238–47. doi: 10.1111/1751-7915.12875 (PMC5743821; doi:10.1111/1751-7915.12875)
Supplement: Supplementary file 1 — Fig. S1. Pristinamycin‐dependent growth of Pip‐ON based dprE1 conditional mutants. [file MBT2-11-238-s001.docx]

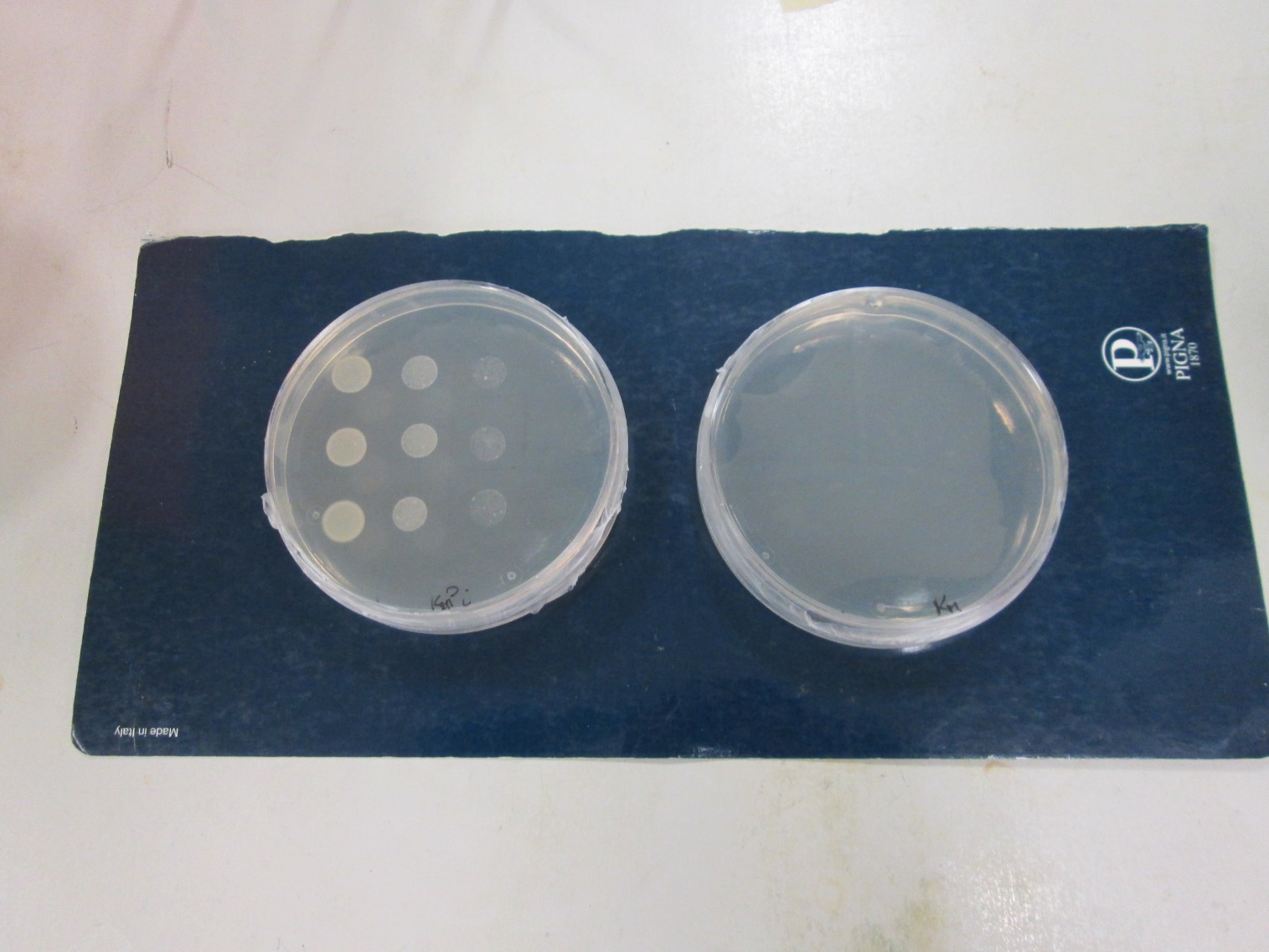


**Pristinamycin**

**TB434**

**TB435**

**TB436**

**No pristinamycin**

**Figure S1.** Pristinamycin-dependent growth of Pip-ON based *dprE1* conditional mutants. Different dilutions of the three conditional mutants were spotted on 7H10 plates with or without 100 ng/ml pristinamycin.
