# Supplementary material for: Promoter mutagenesis for fine‐tuning expression of essential genes in Mycobacterium tuberculosis
Source: Microb Biotechnol. 2017 Oct 27;11(1):238–47. doi: 10.1111/1751-7915.12875 (PMC5743821; doi:10.1111/1751-7915.12875)
Supplement: Supplementary file 2 — Fig. S2. Strategy to construct promoter mutants. [file MBT2-11-238-s002.docx]

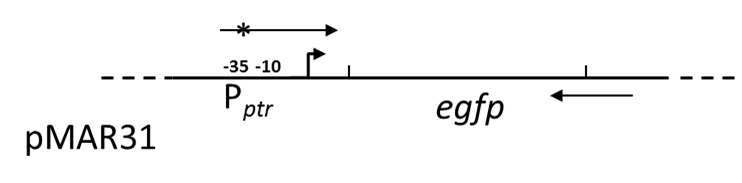


**Figure S2.** Strategy to construct promoter mutants. The P*_ptr_* –*egfp* fragment contained in pMAR31 was PCR-amplified with forward primers containing the mutations and a reverse primer complementary to the end of *egfp*. Straight arrows: primers; asterisk: single point mutation.
