# Supplementary material for: Promoter mutagenesis for fine‐tuning expression of essential genes in Mycobacterium tuberculosis
Source: Microb Biotechnol. 2017 Oct 27;11(1):238–47. doi: 10.1111/1751-7915.12875 (PMC5743821; doi:10.1111/1751-7915.12875)
Supplement: Supplementary file 3 — Table S1. Mutagenized promoters and corresponding strains. [file MBT2-11-238-s003.docx]

| **Table S1. Mutagenized promoters and corresponding strains** | | | | | | | | |
| --- | --- | --- | --- | --- | --- | --- | --- | --- |
|  | Promoter region | Original sequence | Mutagenized sequence | Integrative plasmid | *Msm* strains | *Mtb* strains | Replicative plasmid | *Mtb* strains |
|  |  |  |  |  |  |  |  |  |
|  | wt promoter |  |  | pAGN41 | MS200 | TB263 | pAGN59 | TB272 |
| 1 | Spacer | gcgtatgggaatctctt | **t**gcgtatgggaatctctt | pFRA120 | MS217 | TB267 | pAGN51 | TB273 |
| 2 | Spacer | gcgtatgggaatctctt | **tg**gcgtatgggaatctctt | pFRA123 | MS218 | TB300 |  |  |
| 3 | -35 | **t**GTACa | **c**GTACa | pFRA116 | MS219 | TB264 | pAGN53 | TB275 |
| 4 | -35 | tGTAC**a** | tGTAC**g** | pFRA117 | MS220 | TB291 |  |  |
| 5 | -35 | **t**GTAC**a** | **c**GTAC**g** | pFRA121 |  | TB315 | pAGN55 | TB277 |
| 6 | -35 | **t**GTACa | **g**GTACa | pFRA118 | MS222 | TB265 |  |  |
| 7 | -35 | tGTAC**a** | tGTAC**c** | pFRA119 | MS223 | TB266 |  |  |
| 8 | -35 | **t**GTAC**a** | **g**GTAC**c** | pFRA122 |  | TB316 |  |  |
| 9 | -10 | GTAC**g**gt | GTAC**a**gt | pAGN85 | MS231 | TB292 |  |  |
| 10 | -10 | GTACg**g**t | GTACg**a**t | pAGN86 | MS232 | TB293 |  |  |
| 11 | -10 | GTACgg**t** | GTACgg**c** | pAGN87 | MS233 | TB294 |  |  |
| 12 | -10 | GTAC**ggt** | GTAC**aac** | pAGN88 | MS234 | TB295 |  |  |
| 13 | -10 | GTAC**g**gt | GTAC**t**gt | pAGN89 | MS235 | TB296 |  |  |
| 14 | -10 | GTACg**g**t | GTACg**t**t | pAGN90 | MS236 | TB297 |  |  |
| 15 | -10 | GTACgg**t** | GTACgg**g** | pAGN91 | MS237 | TB298 | pAGN83 | TB287 |
| 16 | -10 | GTAC**ggt** | GTAC**ttg** | pAGN92 | MS238 | TB299 |  |  |
