# Supplementary material for: Promoter mutagenesis for fine‐tuning expression of essential genes in Mycobacterium tuberculosis
Source: Microb Biotechnol. 2017 Oct 27;11(1):238–47. doi: 10.1111/1751-7915.12875 (PMC5743821; doi:10.1111/1751-7915.12875)
Supplement: Supplementary file 4 — Table S2. Primers used in this study. [file MBT2-11-238-s004.docx]

| **Table S2. Primers used in this study** | | |
| --- | --- | --- |
| **Primer** | **Sequence 5’🡪3’** | **Description** |
| RP1335 | tacatgcatgtgagcaagggcgaggag | *egfp* amplification from pMV4-36 |
| RP1336 | caggcctttacttgtacagctcgtccat |  |
| RP1338 | tctagattacttgtacagctcgtccatgccgagag | Reverse primer for PCR-mutagenesis mutations 1🡪 8, XbaI |
| RP1337 | ccgcggagaaatagcgctgtacatgcgtatg | Forward primer for PCR-mutagenesis, SacII; spacer region (mutation 1) |
| RP1339 | ccgcggagaaatagcgctgtaca**TG**gcgtatg | Forward primer for PCR-mutagenesis, SacII; spacer region (mutation 2) |
| RP1340 | ccgcggagaaatagcgc**C**gtacagcgtatg | Forward primer for PCR-mutagenesis, SacII; -35 region modification (mutation 3: T🡪C) |
| RP1341 | ccgcggagaaatagcgctgtacGgcgtatg | Forward primer for PCR-mutagenesis, SacII; -35 region modification (mutation 4: A🡪G) |
| RP1342 | ccgcggagaaatagcgc**C**gtac**G**gcgtatg | Forward primer for PCR-mutagenesis, StuI; -35 region modification (mutation 5: T🡪C, A🡪G) |
| RP1343 | ccgcggagaaatagcgc**G**gtacagcgtatg | Forward primer for PCR-mutagenesis, StuI; -35 region modification (mutation 6: T🡪G) |
| RP1344 | ccgcggagaaatagcgctgtacCgcgtatg | Forward primer for PCR-mutagenesis, StuI; -35 region modification (mutation 7: A🡪C) |
| RP1345 | ccgcggagaaatagcgc**G**gtac**C**gcgtatg | Forward primer for PCR-mutagenesis, StuI; -35 region modification (mutation 8: T🡪G, A🡪C) |
| RP1347 | tctagattacttgtacagctcgtccatgccgagagtgatcccggc | Reverse primer for PCR-mutagenesis mutations 9🡪 16, XbaI |
| RP1346 | ccgcggagaaatagcgctgtacagcgtatgggaatctcttgtac**A**gtgtacgagt | Forward primer for PCR-mutagenesis, StuI; -10 region modification (mutation 9: G🡪A) |
| RP1348 | ccgcggagaaatagcgctgtacagcgtatgggaatctcttgtacgAtgtacgagt | Forward primer for PCR-mutagenesis, StuI; -10 region modification (mutation 10: G🡪A) |
| RP1349 | ccgcggagaaatagcgctgtacagcgtatgggaatctcttgtacgg**C**gtacgagt | Forward primer for PCR-mutagenesis, StuI; -10 region modification (mutation 11: T🡪C) |
| RP1350 | ccgcggagaaatagcgctgtacagcgtatgggaatctcttgtacAACgtacgagt | Forward primer for PCR-mutagenesis, StuI; -10 region modification (mutation 12: GGT🡪AAC) |
| RP1351 | ccgcggagaaatagcgctgtacagcgtatgggaatctcttgtacTgtgtacgagt | Forward primer for PCR-mutagenesis, StuI; -10 region modification (mutation 13: G🡪T |
| RP1352 | ccgcggagaaatagcgctgtacagcgtatgggaatctcttgtacgTtgtacgagt | Forward primer for PCR-mutagenesis, StuI; -10 region modification (mutation 14: G🡪T) |
| RP1353 | ccgcggagaaatagcgctgtacagcgtatgggaatctcttgtacggGgtacgagt | Forward primer for PCR-mutagenesis, StuI; -10 region modification (mutation 15: T🡪G) |
| RP1354 | ccgcggagaaatagcgctgtacagcgtatgggaatctcttgtacTTGgtacgagt | Forward primer for PCR-mutagenesis, StuI; -10 region modification (mutation 16: GGT🡪TTG) |
| RP1629 | acgcgtcataattcgcacaccgcacca | Forward/ reverse primers for amplification of first 518bp upstream *dprE1* |
| Rp1630 | atgcatcgtcaaagagggtacccgcc |  |
| RP1354 | ccgcggagaaatagcgctgtacagcgtatgggaatctcttgtacTTGgtacgagt | Forward primer for PCR-mutagenesis, StuI; -10 region modification GGT🡪TTG |
| RP806 | atgcatttgagcgtgggagctaccactaccgc | *F*orward/ reverse primers for amplification of *dprE1* gene (for conditional mutants construction) |
| RP807 | atcgatctacagcagctccaagcgtcfgggcca |  |
